# Gegen Qinlian Decoction Attenuates Colitis-Associated Colorectal Cancer via Suppressing TLR4 Signaling Pathway Based on Network Pharmacology and In Vivo/In Vitro Experimental Validation

**DOI:** 10.3390/ph18010012

**Published:** 2024-12-25

**Authors:** Yaoyao Xu, Qiaoyan Cai, Chunyu Zhao, Weixiang Zhang, Xinting Xu, Haowei Lin, Yuxing Lin, Daxin Chen, Shan Lin, Peizhi Jia, Meiling Wang, Ling Zhang, Wei Lin

**Affiliations:** 1 Academy of Integrative Medicine, Fujian University of Traditional Chinese Medicine, Fuzhou 350122, China; 15970234177@163.com (Y.X.); cqy2005899@163.com (Q.C.); 13353758983@163.com (C.Z.); 13860822322@163.com (W.Z.); 3220505173@fjtcm.edu.cn (X.X.); 15060120031@sina.cn (H.L.); 18259076221@163.com (Y.L.); lisa3350@163.com (S.L.); Jpz0203@163.com (P.J.); LING39405@126.com (M.W.); 2 College of Integrative Medicine, Fujian University of Traditional Chinese Medicine, Fuzhou 350122, China; 3 Fujian Key Laboratory of Integrative Medicine on Geriatrics, Fujian University of Traditional Chinese Medicine, Fuzhou 350122, China; 4 Innovation and Transformation Center, Fujian University of Traditional Chinese Medicine, Fuzhou 350122, China; cdx1125@126.com

**Keywords:** Gegen Qinlian Decoction, colitis-associated colorectal cancer, azoxymethane/dextran sodium sulfate, inflammatory response, network pharmacology, TLR4 signaling pathway

## Abstract

**Background**: Gegen Qinlian Decoction (GQD), is used for intestinal disorders like ulcerative colitis, irritable bowel syndrome, and colorectal cancer. But the precise mechanisms underlying its anti-inflammatory and anti-tumor effects are not fully elucidated. **Methods**: Use network pharmacology to identify targets and pathways of GQD. In vivo (azoxymethane/dextran sodium sulfate (AOM/DSS)-induced colitis-associated colorectal cancer (CAC) mouse model) and in vitro (lipopolysaccharide (LPS)-stimulated RAW264.7 macrophages) experiments were conducted to explore GQD’s anti-inflammatory and anti-tumor effects. We monitored mouse body weight and disease activity index (DAI), and evaluated colon cancer tissues using hematoxylin and eosin staining. Expression of Ki67 and F4/80 was determined by immunohistochemistry analysis. The protein levels of TLR4 signaling pathway were assessed by western blotting analysis. Enzyme-linked immunosorbent assay measured IL-1β, IL-6, and TNF-α levels. Immunofluorescence (IF) staining visualized NF-κB and IRF3 translocation. **Results**: There were 18, 9, 24 and 77 active ingredients in the four herbs of GQD, respectively, targeting 435, 156, 485 and 691 genes. Through data platform analysis, it was concluded that there were 1104 target genes of GQD and 2022 target genes of CAC. Moreover, there were 99 intersecting genes between GQD and CAC. The core targets of GQD contained NFKB1, IL1B, IL6, TLR4, and TNF, and GQD reduced inflammation by inhibiting the TLR4 signaling pathway. In vivo experiment, GQD increased mouse body weight, lowered DAI scores, while also alleviating histopathological changes in the colon and decreasing the expressions of Ki67 and F4/80 in the AOM/DSS-induced mice. GQD reduced IL-1β, IL-6, and TNF-α levels in the serum and downregulated TLR4, MyD88, and phosphorylation of IκBα, P65, and IRF3 in the colon tissue from AOM/DSS-induced mice. In vitro, GQD suppressed pro-inflammatory cytokines and TLR4 signaling pathway in the LPS-induced RAW264.7 cells, and combined with TAK242, it further reduced the phosphorylation of IκBα, P65. **Conclusions**: GQD mitigated CAC by inhibiting the TLR4 signaling pathway, offering a potential therapeutic approach for CAC management.

## 1. Introduction

Ulcerative colitis (UC) is a chronic inflammatory condition of the colon characterised by abdominal pain, diarrhoea and blood in the stool [1,2]. And hemorrhagic diarrhea, abdominal spastic pain, and weight loss are the most frequent symptoms [3]. Over the past few decades, there has been an increasing trend in the occurrence of UC with the annual incidence of UC ranging from 8.8 to 23.1 per 100,000 person-years in North America, 0.6 to 24.3 per 100,000 person-years in Europe, and 7.3 to 17.4 in Oceania [4]. Colitis-associated colorectal cancer (CAC) is the most serious complication of UC, which accounts for around 15% of all-causes mortality among patients with inflammatory bowel disease [5,6]. It is reported that the risk of CAC could increase to 30% in patients who have suffered from UC for 35 years or more [7]. The risk of CAC is elevated as UC progresses, positively correlating with both the degree of inflammation and the extent of the disease [8]. Patients with UC have an 18–20% higher risk of developing colon cancer compared to the general population, so suppressing the progression of UC is also an important strategy for preventing the development of CAC [9,10].

Indeed, the etiology of CAC is intricately linked to chronic inflammation, which can be persistent or recurrent [11,12,13]. Current research has established the involvement of various Toll-like receptors (TLRs) in modulating inflammatory responses that contribute to cancer progression [14,15,16]. Particularly, Toll-like receptor 4 (TLR4), a key pattern recognition receptor essential for intestinal immunity, is often overexpressed in colonic epithelial cells under inflammatory conditions [17,18,19]. Emerging evidence further suggests that TLR4 could act as a biomarker, potentially promoting the development of CAC [20]. Upon activation, TLR4 initiates a signaling cascade by recruiting adaptor proteins such as myeloid differentiation primary response 88 (MyD88) and Toll interleukin-1 receptor (TlR)-domain-containing adaptor-inducing interferon-β (TRIF), leading to the phosphorylation of nuclear transcription factor-κB (NF-κB) and interferon regulatory factor 3 (IRF3). This process enhances the production of pro-inflammatory cytokines, which in turn, fuels the initiation and progression of CAC [21]. Therefore, suppressing the ensuing inflammatory signaling pathways and TLR4-related inflammatory responses is a promising novel therapy for CAC.

Traditional Chinese medicine (TCM) plays a significant role in the prevention and treatment of CAC [22]. Gegen Qinlian Decoction (GQD), a formula that has been preserved for over 2000 years, originates from the treatise on typhoid fever developed by the renowned Chinese physician Zhang Zhongjing. It primarily consists of the following botanical species: *Pueraria lobata* (Willd.) Ohwi (Gegen), *Scutellaria baicalensis* Georgi (Huangqin), *Coptis chinensis* Franch. (Huanglian), and *Glycyrrhiza uralensis* Fisch. (Gancao) in the proportions of 8:3:3:2 [23]. Clinically, it is frequently employed to treat bacterial dysentery, acute enteritis, CAC, and other diseases [24,25]. GQD has been reported to reduce oxidative stress and inflammation and to enhance intestinal barrier function, thereby treating UC [26]. It modulates the homeostasis of Th17/Treg cells, ameliorating UC induced by DSS in mice [27]. Furthermore, GQD has been shown to modify the intestinal flora and tumor microenvironment, potentially slowing tumor development. We hypothesize that GQD may also impede the progression of CAC by suppressing inflammation. However, there is a paucity of studies on its potential mechanisms of action, which warrants further exploration and investigation.

The azoxymethane/dextran sodium sulfate (AOM/DSS) model, proposed in 1998, is a well-established model for studying colorectal tumors associated with chronic inflammation [28]. Research has demonstrated that tumors developed in AOM/DSS-treated mice closely resemble to human CAC [29]. Therefore, this model is widely used to reveal some of the mechanisms of inflammation-related colon carcinogenesis in the gut [30,31,32]. Macrophages play an essential role in progression of inflammation and lipopolysaccharide (LPS), one of the main components of bacterial cell walls, can induce inflammatory responses in macrophages [33,34]. Therefore, we utilized a CAC mouse model induced by AOM/DSS for the in vivo study and an inflammatory model in RAW264.7 cells stimulated with LPS for the in vitro study to jointly reveal the poteintail mechanisms of action of GQD on CAC.

In our study, we meticulously identified the quality control of GQD using UPLC-MS (Waters, Milford, CT, USA). Network pharmacology was employed to predict targets and pathways of GQD against CAC. An in vivo mouse model of CAC was induced by AOM/DSS, and LPS-induced RAW264.7 cells served as the in vitro model. We initially evaluated the effect of GQD on the symptoms, tumor incidence, and survival of AOM/DSS-induced CAC mice. Subsequently, we further validated the mechanism of GQD in treating CAC, as identified by network pharmacology, through both in vivo and in vitro experiments. Finally, we demonstrated the synergistic effect of GQD and TAK242 in LPS-induced RAW264.7 cells. It is anticipated that these findings will provide ample scientific evidence and an experimental foundation for further studies and the clinical application of GQD in treating CAC patients.

## 2. Results

### 2.1. UPLC-MS Fingerprint Analysis of GQD

**Figure 1:**
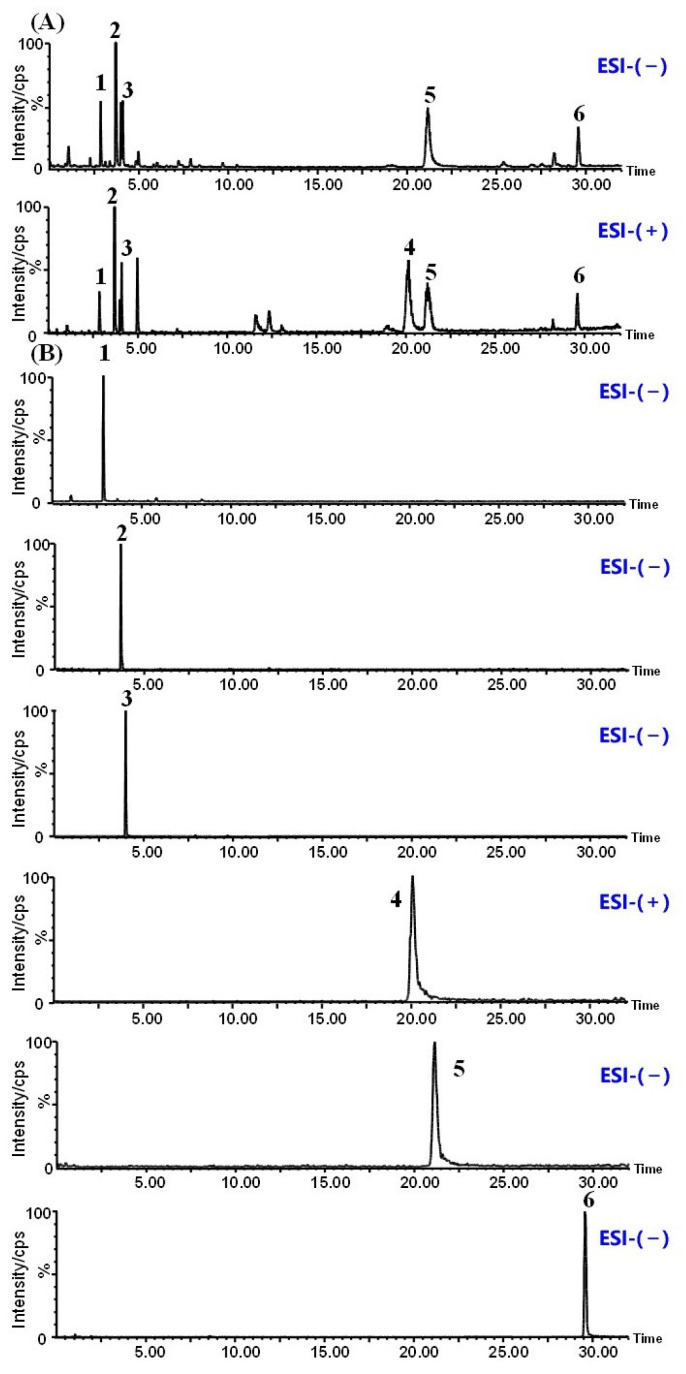
Quality control of GQD using UPLC-MS. (**A**) The positive and negative ion chromatogram of GQD. (**B**) The peaks of reference substances. 1. Puerarin; 2. Liquiritin; 3. Puerarin 6″-O-xyloside; 4. Berberine; 5. Baicalin; 6. Glycyrrhizic acid. GQD: Gegen Qinlian Decoction.

The chromatograms of positive and negative ions for GQD were illustrated in Figure 1A. The reference substances’ peaks of puerarin; liquiritin; puerarin 6″-O-xyloside; berberine; baicalin; and glycyrrhizic acid were identified in Figure 1B, respectively. The results suggested that GQD contained puerarin; liquiritin; puerarin 6″-O-xyloside; berberine; baicalin; and glycyrrhizic acid, supporting a method for controlling the quality of GQD.

### 2.2. Active Ingredients and Target Genes of GQD

**Figure 2:**
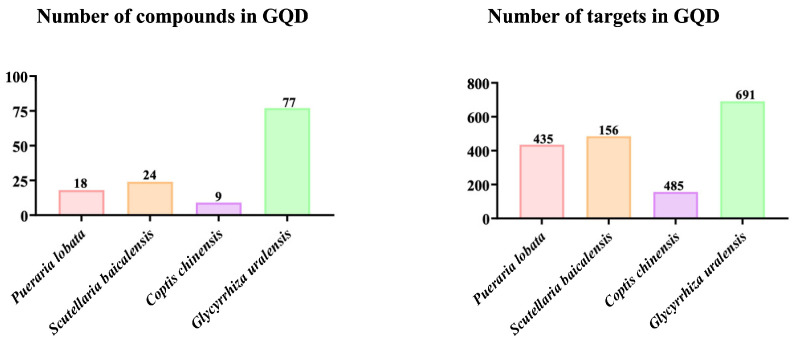
Active ingredients and target genes of GQD. Searching in the Batman-TCM database revealed compounds and target gene information for *Pueraria lobata* (Willd.) Ohwi, *Scutellaria baicalensis* Georgi, *Coptis chinensis* Franch, and *Glycyrrhiza uralensis* Fisch, with specific numbers listed. GQD: Gegen Qinlian Decoction.

Using the Batman-TCM database, the numbers of active ingredients in GQD’s 4 herbs (*Pueraria lobata* (Willd.) Ohwi, *Scutellaria baicalensis* Georgi, *Coptis chinensis* Franch, and *Glycyrrhiza uralensis* Fisch), were 18, 9, 24, and 77, and the corresponding numbers of target genes were 435, 156, 485, and 691, respectively (Figure 2).

### 2.3. Identifying the Intersecting Genes Between GQD and CAC as Well as Creating the Compound-Intersecting Genes-Disease Association Map

**Figure 3:**
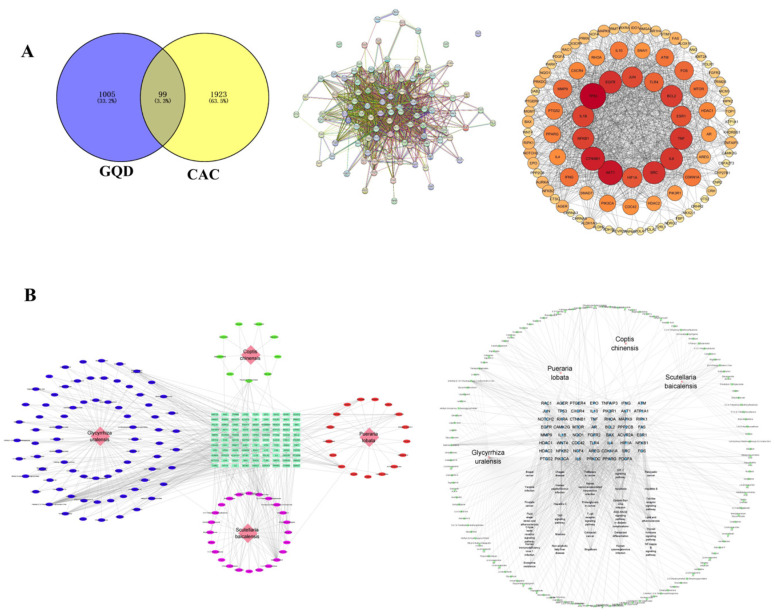
Identifying the intersecting genes between GQD and CAC as well as creating the compound-intersecting genes-disease association map. (**A**) Venny diagram of intersecting genes of GQD and CAC; Analysis of intersecting genes via protein-protein interaction network. (**B**) Development of the compound-targets network and compound-targets-pathway network from the 4 herbs of GQD. GQD: Gegen Qinlian Decoction; CAC: Colitis-Associated Colorectal Cancer.

A total of 2022 CAC disease target genes were acquired from the GeneCards database and the OMIM database. Meanwhile, 1104 target genes of GQD were obtained from the Batman-TCM database. The data obtained from GQD and CAC were analyzed for a total of 99 intersecting genes. Using the intersecting genes to draw the Venny diagram and PPI network map, the findings indicated that the PPI network comprised intersecting genes that consisted of 99 nodes and 1092 edges. Afterward, the original PPI information was input into Cytoscape 3.9.1 for visualization processing (Figure 3A). In the visualized PPI network, a higher degree value of a specific target protein corresponded to a larger node size. This indicated that the protein played a more crucial role in the treatment of CAC with GQD as it interacted with other targets. Furthermore, Cytoscape 3.9.1 was used to develop 4 herbs’ compound-targets network as well as compound-targets-pathway network. The herbs’ compound-targets network was enriched with key inflammatory mediators such as IL-1β, IL-6, TNF, and NF-κB, and was closely related to Toll-like receptor, TNF, and NF-κB related signaling pathways (Figure 3B). It indicated that the GQD may play a therapeutic role in the AOM/DSS-induced CAC mouse model through mediating related inflammatory mediators, and Toll-like receptor signaling pathways.

### 2.4. KEGG and GO Pathway Enrichment Analyses

**Figure 4:**
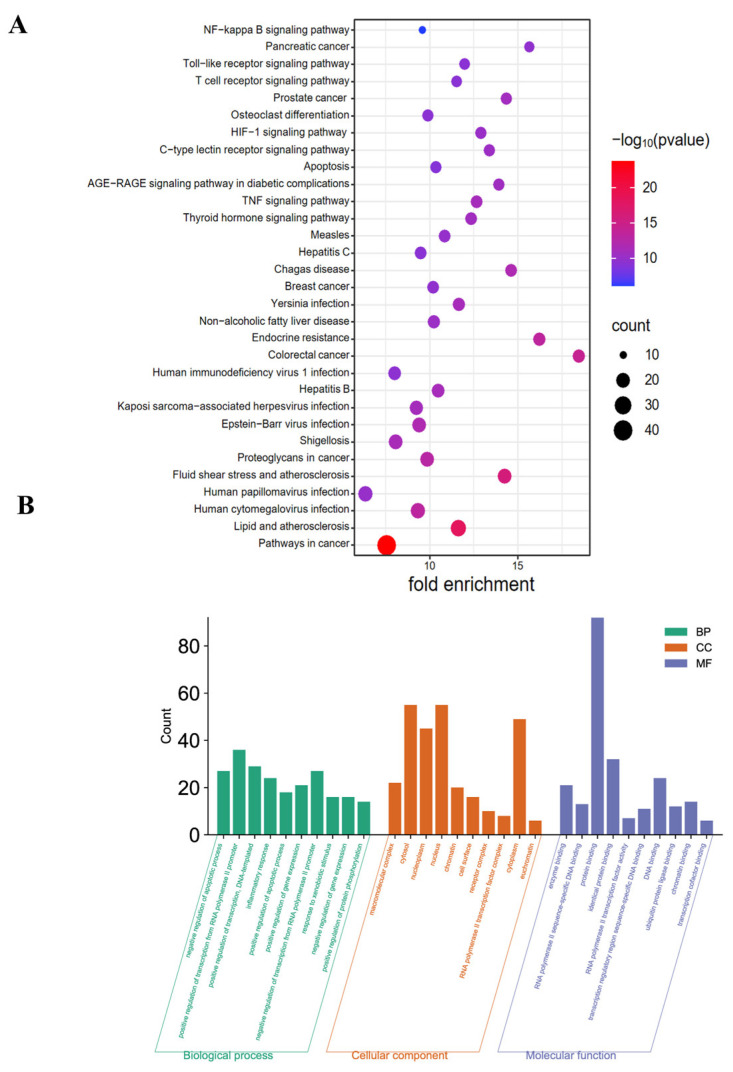
KEGG and GO pathway enrichment analyses. (**A**) Analyzing the pathway enrichment of the intersecting genes between GQD and CAC. (**B**) GO analysis of biological processes, cellular components, and molecular functions associated with the therapeutic effects of GQD for CAC. GQD: Gegen Qinlian Decoction; CAC: Colitis-Associated Colorectal Cancer; KEGG: Kyoto Encyclopedia of Genes and Genomes; GO: Gene Ontology.

To evaluate the potential role of GQD on CAC in more detail, KEGG and GO enrichment analyses were performed on 99 intersecting genes. The results revealed the top-ranking enriched signaling pathways, including the NF-κB signaling pathway, Toll-like receptor signaling pathway, and TNF-α signaling pathway, which were implicated in the progression of CAC according to KEGG analysis (Figure 4A). GO enrichment analysis indicated that the primary biological processes were associated with the inflammatory response. Cellular components were predominantly expressed in the cytoplasm, nucleoplasm, nucleus, chromatin, and cell surface. Molecular functions included positive regulation of protein binding among others (Figure 4B).

### 2.5. GQD Attenuated the Symptoms in AOM/DSS-Induced CAC Mice

**Figure 5:**
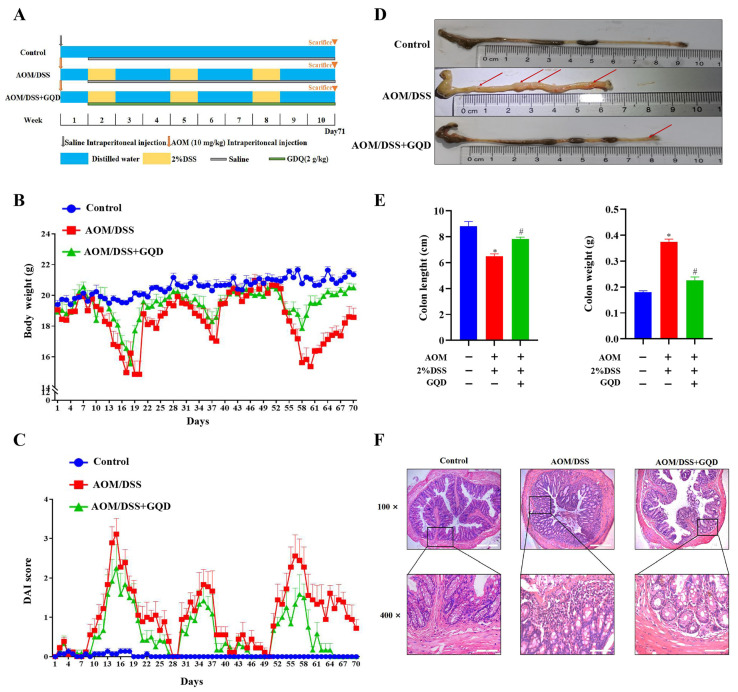
GQD attenuated the symptoms in AOM/DSS-induced CAC mice. (**A**) Sketch of the animal experimental design. (**B**) Body weight of three groups. n = 6. (**C**) The DAI score of three groups. n = 6. (**D**,**E**) The macroscopic pathology, as well as the length and weight of the mouse colon in three groups. The red arrow indicated the location of the tumor. n = 3. (**F**) The pathological morphology of the colon with different multiples via HE staining in three groups. n = 4. * *p* < 0.05 vs. Control group; ^#^ *p* < 0.05 vs. AOM/DSS group. GQD: Gegen Qinlian Decoction; CAC: Colitis-Associated Colorectal Cancer; AOM/DSS: Azoxymethane/Dextran Sodium Sulfate; DAI: Disease Activity Index; HE: Hematoxylin and Eosin.

To investigate the possible effect of GQD on CAC, the AOM/DSS-induced CAC mouse model was established. The detailed modeling scheme is shown in Figure 5A. As illustrated in Figure 5B, the body weight of the mice in the AOM/DSS group was significantly reduced in comparison to the Control group; however, the weight loss was reduced by GQD. The result shown in Figure 5C indicated that the disease activity index (DAI) score was elevated in the AOM/DSS group in comparison to the Control group, and it was reduced in the AOM/DSS + GQD group. As depicted in Figure 5D,E, in contrast to the Control group, the colon length of mice in the AOM/DSS group was greatly shorter and their weight was increased; however, GQD administration reversed the phenomenon. Hematoxylin and eosin (HE) staining found that the colonic mucosa of mice in the Control group was undamaged, with the glands arranged neatly, without inflammatory cell infiltration, smooth surface, and no congestion and edema; the colonic tissue of mice in the AOM/DSS group had crypt destruction, disordered glandular arrangement, and a significant number of inflammatory cell infiltration and reduced cup cells. After GQD intervention, the degree of colonic epithelial mucosal erosion was significantly reduced, the number of normal glands was significantly increased, and inflammatory infiltrating cells were significantly reduced (Figure 5F). Taken together, these results suggested that GQD can significantly lessen the symptoms of AOM/DSS-induced CAC mice.

### 2.6. GQD Reduced the Tumor Incidence and Improved the Survival of AOM/DSS-Induced CAC Mice

**Figure 6:**
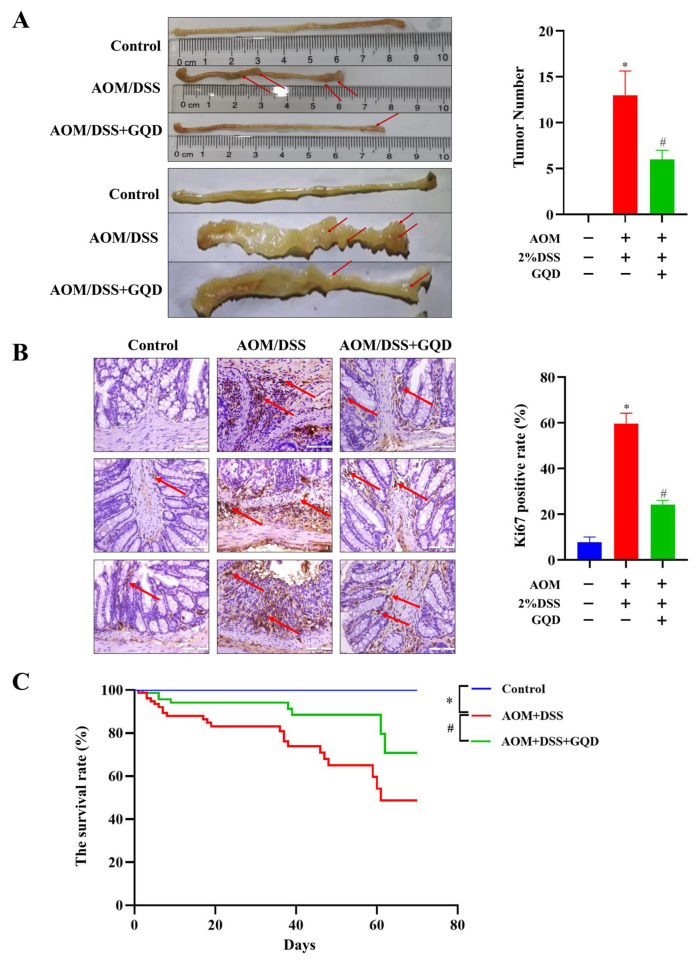
GQD reduced the tumor incidence and improved the survival rate in AOM/DSS-induced CAC mice. (**A**) Comparison of colonic macroscopic morphology and number of tumors in three groups. The red arrow indicated the location of the tumor. n = 3. (**B**) The IHC staining results of Ki67 in three groups. The red arrow represented the characteristics of Ki67 positive expression. n = 4. (**C**) The survival rate of the three groups. n = 6. * *p* < 0.05 vs. Control group; ^#^ *p* < 0.05 vs. AOM/DSS group. GQD: Gegen Qinlian Decoction; CAC: Colitis-Associated Colorectal Cancer; AOM/DSS: Azoxymethane/Dextran Sodium Sulfate; IHC: Immunohistochemistry.

The colon carcinogenesis was investigated by counting the tumor number. As depicted in Figure 6A, the tumor number was 0 in the Control group, while the tumor number displayed a significant increase after treatment with AOM/DSS, and the tumors adhered to the colon tissue, and the surface was more obviously congested and edematous, indicating that the modelling was successful. However, in comparison with the AOM/DSS group, the number of tumors was substantially reduced in the AOM/DSS + GQD group. In hyperplasia and tumor lesions, the expression of Ki67 protein was increased [35]. Therefore, we used the immunohistochemistry (IHC) analysis to determine the expression of tumor marker Ki67 in three groups. As illustrated in Figure 6B, the positive rate of Ki67 in the AOM/DSS group was considerably elevated than that in the Control group, nevertheless, GQD could reverse its positive rate in the AOM/DSS-treated mice. Compared with the Control group, the survival rate in the AOM/DSS group was significantly decreased. However, this phenomenon was reversed after treatment with GQD (Figure 6C). Collectively, this finding suggested that GQD had an inhibitory effect on tumor growth and enhanced the survival rate in AOM/DSS-induced CAC mice.

### 2.7. GQD Inhibited Inflammation by Downregulation of the TLR4-Related Signaling Pathways in AOM/DSS-Induced CAC Mice

**Figure 7:**
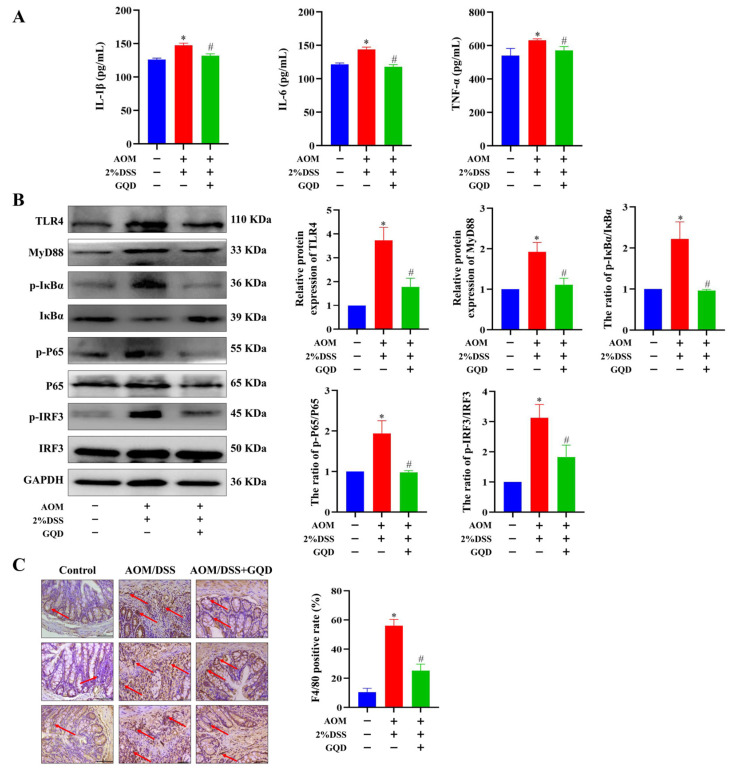
GQD inhibited inflammation by downregulating the TLR4-related signaling pathways in AOM/DSS-induced CAC mice. (**A**) Serum inflammatory factors in three groups. n = 4. (**B**) The protein expression of TLR4, Myd88, p-IκBα, IκBα, p-P65, P65, p-IRF3, and IRF3 in three groups. n = 3. (**C**) The IHC staining results of F4/80 in three groups. The red arrow represented the characteristics of F4/80 positive expression. n = 4. * *p* < 0.05 vs. Control group; ^#^ *p* < 0.05 vs. AOM/DSS group. GQD: Gegen Qinlian Decoction; CAC: Colitis-Associated Colorectal Cancer; AOM/DSS: Azoxymethane/Dextran Sodium Sulfate; IHC: Immunohistochemistry.

Recent research has provided evidence that inflammation plays a great role in the progression and development of various types of cancer [36]. Network pharmacology results showed that GQD might have outstanding advantages in inhibiting inflammatory responses. Therefore, we first investigated whether GQD could inhibit the inflammatory response in AOM/DSS-induced CAC mice. As depicted in Figure 7A, the levels of inflammatory mediators (IL-1β, IL-6 and TNF-α) were greatly increased in the serum of AOM/DSS group in contrast to the Control group. While the AOM/DSS + GQD group showed a reduction in inflammatory factors. An analysis of KEGG enrichment revealed that the TLR4 signaling pathway was significantly involved in the regulation of CAC treated with GQD. As a pivotal member of the TLR family and a prototypical inflammatory mediator, TLR4 serves as an intermediary linking innate immunity to adaptive immunity and infection to inflammation [37]. Subsequently, the western blotting analysis was carried out to examine the proteins of TLR4-related pathways in three groups. The results demonstrated that the AOM/DSS-treated mice had a higher expression of TLR4, MyD88, and the ratios of p-IκBα/IκBα, p-P65/P65 and p-IRF3/IRF3, whereas GQD significantly suppressed them (Figure 7B). To further evaluate the macrophage infiltration, IHC analysis was performed to determine the expression of its marker F4/80 in three groups. As shown in Figure 7C, it was found that the positive rate of F4/80 was greatly high in the AOM/DSS group, which was considerably reduced after GQD treatment. Collectively, these findings suggested that GQD could potentially inhibit the progression of CAC by decreasing the TLR4-related NF-κB and IRF3 signaling pathways, and then block the release of inflammatory factors.

### 2.8. GQD Reduced the Inflammatory Cytokine Secretion in LPS-Induced RAW264.7 Cells

**Figure 8:**
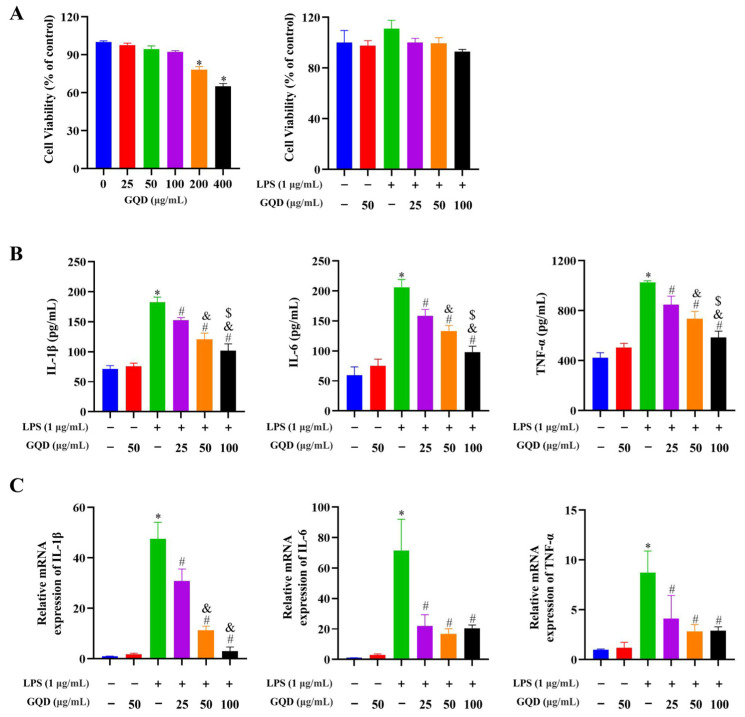
GQD inhibited the inflammatory cytokine secretion in LPS-induced RAW264.7 cells. (**A**) The impact of GQD on the viability of RAW264.7 cells via the CCK8 assay. n = 6. (**B**,**C**) The levels of IL-1β, IL-6, and TNF-α in each group. n = 3. * *p* < 0.05 vs. Control group; ^#^ *p* < 0.05 vs. LPS group; ^&^ *p* < 0.05 vs. LPS + 25 μg/mL GQD group; ^$^ *p* < 0.05 vs. LPS + 50 μg/mL GQD group. GQD: Gegen Qinlian Decoction; LPS: Lipopolysaccharide.

The above-mentioned experimental findings suggested that GQD greatly inhibited macrophage infiltration and inhibited inflammation-associated signaling pathways in the AOM/DSS-treated mice. To elucidate the underlying mechanisms, we utilized an in vitro model of LPS-induced inflammation in RAW264.7 cells. Firstly, the concentrations of GQD with less than 100 µg/mL had no cytotoxicity to RAW264.7 cells with or without 1 µg/mL LPS (Figure 8A). Therefore, the concentrations of 25, 50, and 100 µg/mL GQD were used in the following experiments. Secondly, both enzyme-linked immunosorbent assay (ELISA) and reverse transcription-quantitative polymerase chain reaction (RT-qPCR) analysis demonstrated that the levels of IL-1β, IL-6, and TNF-α greatly increased in the LPS-induced RAW264.7 cells, which was reversed after GQD intervention (Figure 8B,C). Collectively, these findings demonstrated that GQD possessed an anti-inflammatory effect.

### 2.9. GQD Inhibited TLR4-Related Signaling Pathways in LPS-Induced RAW264.7 Cells

**Figure 9:**
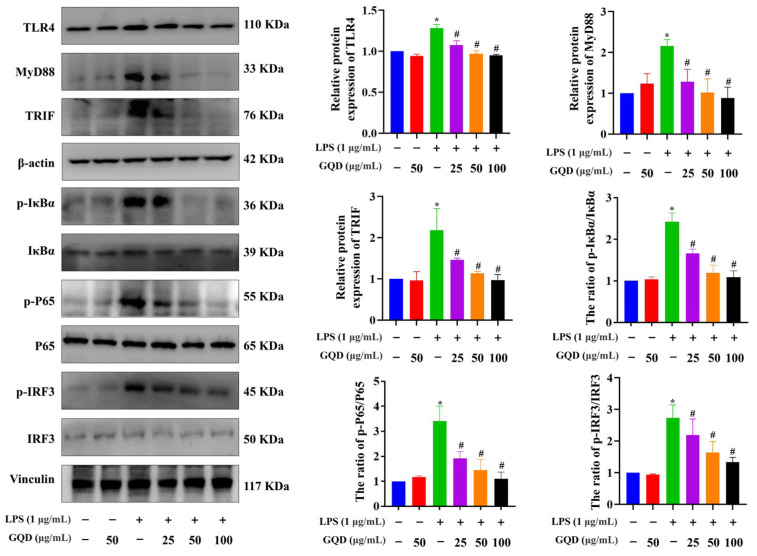
GQD inhibited TLR4-ralated signaling pathway in LPS-induced RAW264.7 cells. The protein expression of TLR4, Myd88, TRIF, p-IκBα, IκBα, p-P65, P65, p-IRF3, IFR3 in each group. n = 3. * *p* < 0.05 vs. Control group; ^#^ *p* < 0.05 vs. LPS group. GQD: Gegen Qinlian Decoction; LPS: Lipopolysaccharide.

To further study potential regulatory effects of GQD on inflammation-related signaling pathways in macrophages, western blotting analysis was carried out to assess the levels of TLR4, MyD88, TRIF, p-IκBα, IκBα, p-P65, P65, p-IRF3 and IRF3 in each group. The results depicted that the level of TLR4, MyD88, TRIF, and the ratios of p-IκBα/IκBα, p-P65/P65, p-IRF3/IRF3 were greatly increased in the LPS-induced RAW264.7 cells relative to the Control group. The expressions of TLR4, MyD88, TRIF, and as well as the ratios of p-IκBα/IκBα, p-P65/P65, and p-IRF3/IRF3 were all significantly reduced following treatment with GQD (Figure 9). Overall, GQD can impede the downstream signaling pathways of TLR4.

### 2.10. GQD Inhibited Nuclear Translocation of NF-κB, IRF3 in LPS-Induced RAW264.7 Cells

**Figure 10:**
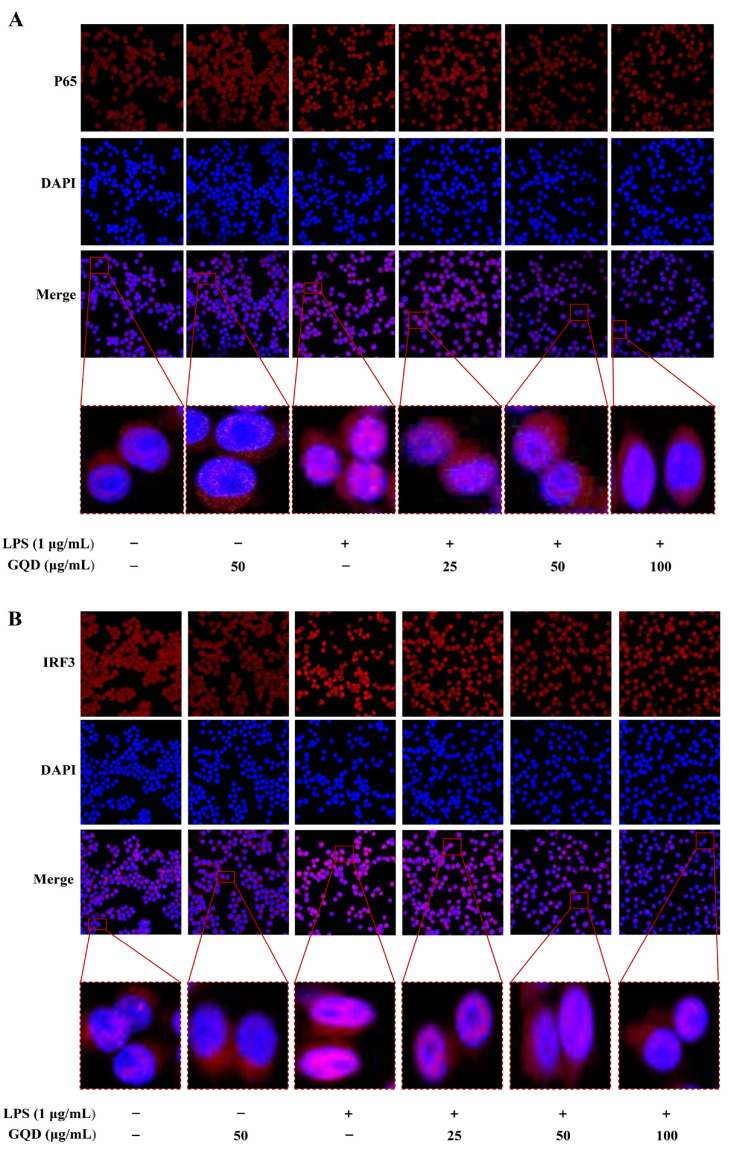
GQD inhibited nuclear translocation of NF-κB, IRF3 in LPS-induced RAW264.7 cells. (**A**) The expression and nuclear translocation of NF-κB protein in each group by IF staining. n = 4. (**B**) The expression and nuclear translocation of IRF3 protein in each group by IF staining. n = 4. GQD: Gegen Qinlian Decoction; LPS: Lipopolysaccharide; IF: Immunofluorescence.

NF-κB is known to be a major transcriptional regulator whose activation induces transcription of pro-inflammatory factors, and IRF3, a significant transcription factor in the innate immune response, and there are research reports that knocking out IRF3 can reduce the occurrence of inflammatory reactions [38,39]. Therefore, we investigated the role of GQD in inhibiting nuclear translocation of NF-κB and IRF3. As depicted in Figure 10A,B, in contrast to the Control group, most of the red fluorescent representing NF-κB and IRF3 transferred from the cytoplasm to the nucleus in the LPS-induced RAW264.7 cells. However, GQD treatment decreased the nuclear translocation of NF-κB and IRF3. The result indicated that GQD exerted a key influence in the inhibition of NF-κB, IRF3 nuclear translocation.

### 2.11. The Combination of GQD and TAK242 Was Synergistic to Suppress the TLR4-Related Signaling Pathways

**Figure 11:**
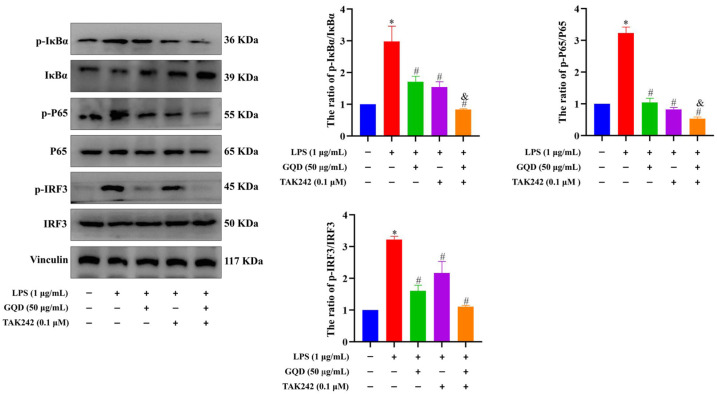
Synergistic effect of GQD and TAK242 on the inhibiting TLR4-related signaling pathways. The protein expression of p-IκBα, IκBα, p-P65, P65, p-IRF3, IFR3 in each group. n = 3. * *p* < 0.05 vs. Control group; ^#^ *p* < 0.05 vs. LPS group; ^&^ *p* < 0.05 vs. LPS + GQD. GQD: Gegen Qinlian Decoction; LPS: Lipopolysaccharide.

To substantiate the remarkable effects of GQD on the TLR4-related signaling pathways, we treated LPS-induced RAW264.7 cells with TLR4 inhibitor TAK242 and GQD. Western blotting analysis was used to determine the protein levels of p-IκBα, IκBα, p-P65, P65, p-IRF3, and IRF3 in each group. The data indicated that the LPS + GQD, LPS + TAK242, and LPS + TAK242 + GQD groups markedly decreased the ratio of p-IκBα/IκBα, p-P65/P65, and p-IRF3/IRF3 compared with the LPS group. Meanwhile, TAK242 and GQD exemplified a synergistic effect on the ratio of p-IκBα/IκBα, p-P65/P65 (Figure 11). This strongly suggested that GQD worked synergistically with TAK242 to suppress inflammation via downregulating TLR4-related signaling pathways.

## 3. Discussion

Our research showed that GQD had a positive influence on suppressing inflammatory responses in the gut, as well as anti-tumorigenesis, and improving survival rate of the AOM/DSS-treated mice. Network pharmacology predictions had underscored the TLR4 signaling pathway as a pivotal conduit through which GQD could exert its anti-neoplastic effects. In vitro assays showed that GQD significantly suppressed the stimulation of NF-κB and IRF3 of TLR4-related signaling pathways in LPS-induced RAW264.7 cells, preventing the entry of NF-κB and IRF3 into the nucleus, there by suppressing the synthesis of pro-inflammatory factors and effectively showed a synergistic therapeutic impact when combined with TAK242. With a venerable clinical history, GQD has been extensively applied in the treatment of intestinal disorders, including UC, irritable bowel syndrome, and colorectal cancer [40,41,42]. The active constituents within GQD, such as puerarin, daidzein, baicalein, baicalin, berberine, palmatine hydrochloride, and glycyrrhizic acid, have been identified as key players in the combat against colon adenocarcinoma [43]. The diverse pharmacological properties of *Pueraria lobata* (Willd.) Ohwi, like antioxidant, cancer-resisting, anti-inflammatory, and analgesic effects, may account for its therapeutic benefits in various medical applications [44]. Berberine is the major constituent of *Coptis chinensis* Franch and its pharmacological effects range from anti-inflammatory to antioxidant, antibacterial, antiviral, and anticancer virtues [45]. The primary pharmaceutical constituent found in *Scutellaria baicalensis* Georgi is baicalin, which exhibits potent anti-inflammatory and antioxidant properties [46,47]. Glycyrrhizin, one of the primary chemical components in *Glycyrrhiza uralensis* Fisch, exhibits anti-inflammatory and anti-colon cancer effects by reducing hypertrophic tumor cell infiltration [48]. These studies provide powerful evidence for GQD’s efficacy against tumorigenesis in CRC models.

Inhibition of inflammatory response is an important step in the treatment of CAC. Our research had confirmed that GQD improved the survival rate of mice with AOM/DSS-induced colon cancer. It reduced the severity of colon inflammation, as indicated by lower DAI scores, and improved colon tissue morphology. GQD also decreased the concentration of inflammatory mediators in serum. Moreover, it inhibited the growth of colon tumors. These effects were attributed to the modulation of TLR4-associated pathways, such as NF-κB and IRF3. Overall, our findings suggested that GQD had therapeutic potential for treating CAC by improving the inflammatory microenvironment in colon tissues and slowing down the progression of inflammation-cancer transformation. The inflammatory response catalyzes the progression of cancer, and up to 25% of human malignancies are associated with chronic inflammation and infection [49]. All patients with inflammatory bowel disease exhibit an of pro-inflammatory factors, and the majority of colon cancers studied to date have been linked to an inflammatory microenvironment. The results of in vivo experimental results showed that GQD prevent the development of CAC, at least in part, by suppressing abnormal inflammatory responses.

TLR4 is a pivotal pattern recognition receptor in innate immunity, playing a crucial role in mediating inflammatory responses [50]. Knockdown of TLR4 using the antagonist TLR4 antibody, CAC induced by AOM and DSS decreased significantly [51]. Using the network pharmacological method, the mechanism of GQD against colon cancer was revealed. Analysis shows that GQD has 99 core targets in CAC and may inhibit CAC by inhibiting the inflammatory response via the TLR4-related signaling pathways. One part of the infection response in various cell types is IRF3. Verified the NF-κB and IRF3 pathways are related to the IκB kinase [52]. These two signaling pathways have an interaction with genes on the promotor level [53]. Alongside the function of the innate immune response, it depends on collaboration between the NF-κB and IRF3 signaling pathways, which are highly correlated [54,55,56,57]. In vitro cellular experiments further demonstrated the significant anti-inflammatory efficacy of GQD and the potential curative effect was related to the inhibition of TLR4-associated signaling pathways like NF-κB and IRF3. Furthermore, in vitro experiments, we found that the combination of GQD and TAK242 had a synergistic effect and both of them significantly down-regulated the expression levels of phosphorylated IκBα and NF-κB in LPS-induced RAW264.7 cells. These findings robustly established GQD’s ability to suppress TLR4-related signaling cascades, thereby reducing inflammation and retarding the progression of CAC.

This subject needed further study, even though explored the therapeutic effect of GDQ on CAC, but did not directly verify its inhibitory effect on colorectal cancer itself. It is suggested that future research could use AOM to first establish a colorectal cancer model, followed by drug intervention to assess the inhibitory effect of GQD. Furthermore, this study only used macrophages for in vitro experiments and did not involve tumor cells. Future research could expand to co-culture of macrophages and tumor cells to further explore the mechanism of GQD.

## 4. Materials and Methods

### 4.1. Chemicals and Reagents

Azoxymethane (A5486) was purchased from Sigma-Aldrich (St. Louis, MO, USA); DSS (216011090) was purchased from MP Biomedicals (Irvine, CA, USA); TAK-242 (CLI-095) was obtained from MedChemExpress (New Jersey, USA); Puerarin (T2815), liquiritin (T2899), puerarin 6″-O-xyloside (T5S2083), berberine (T4S0797), baicalin (T2775), glycyrrhizic acid (T2741) were purchased from TargetMol Chemicals Inc. (Shanghai, China). *Pueraria lobata* (Willd.) Ohwi (C442220601), *Scutellaria baicalensis* Georgi (C130220702), *Coptis chinensis* Franch (C129220702), and *Glycyrrhiza uralensis* Fisch (C118220701) were purchased from Hebei Anjia Pharmaceutical Co., Ltd. (Anguo, China); Ki67 antibody (AB16667) was purchased from Abcam (Cambridge, UK); IRF3 (11904) and p-IRF3 (29047S) antibodies were purchased from the Cell Signaling Technology (Beverly, MA, USA); TLR4 (66350-1-Ig), MyD88 (23230-1-AP), TRIF (23288-1-AP), β-actin (66009-1-Ig), GAPDH (60004-1-Ig), Vinculin (66305-1-Ig), HRP-conjugated Goat Anti-Mouse IgG (H+L) (SA00001-1), HRP-conjugated Goat Anti-Rabbit IgG (H+L) (SA00001-2), F4/80 (28463-1-AP) antibodies were all purchased from Proteintech (Wuhan, China). p-IκBα (ABP0038), IκBα (ABP57568), p-P65 (ABP0043), P65 (ABM40111) antibodies were purchased from Abbkine Biotechnology Co., Ltd. (Wuhan, China). Cole’s Hematoxylin Solution (G1140), Eosin Y Stain Solution (G1100) and CCK8 kit (CA1210) were purchased from Solaibao Technology Co., Ltd. (Beijing, China); Immunohistochemical kit (Kit-0017) was purchased from Fuzhou MaiNew Biotechnology Development Co., Ltd. (Fuzhou, China); Fecal occult blood (OB) reagent (BA2020B) was purchased from Zhuhai Beso Biotechnology Co., Ltd. (Zhuhai, China); HiScript 1st Strand cDNA Synthesis kit (R211-02) was purchased from Nanjing Novizan Biotechnology Co., Ltd. (Nanjing, China); Trizol (15596026CN) and SYBR^®^ Select Master Mix kit (4472908) was purchased from Thermo Fisher Scientific (Waltham, MA, USA); ELISA kit mouse IL-1β (MM-0040M1), mouse IL-6 (MM-0163M1), mouse TNF-α (MM-0132M1) were procured from JiangSu enzyme free Industrial Co., Ltd. (Nanjing, China).

### 4.2. Preparation of GQD Extractions

GQD is composed of 4 herbs: *Pueraria lobata* (Willd.) Ohwi (24 g), *Scutellaria baicalensis* Georgi (9 g), *Coptis chinensis* Franch (9 g), and *Glycyrrhiza uralensis* Fisch (6 g). To prepare the GQD extract, the herbs were initially soaked in distilled water for 30 min at ambient temperature. Subsequently, the mixture was subjected to reflux extraction, which was performed three times, with each extraction lasting 1 h using an eightfold volume (*w*/*v*) of distilled water relative to the weight of the herbs. After the extraction process was completed, the combined decoctions were concentrated using a rotary evaporator to obtain the extract, and the extract was then freeze-dried and ground into powder for in vivo and in vitro experiments. Approximately 48 g of GQD’s formula could yield 15.38 g of GQD power.

### 4.3. Quality Control of GQD Using UPLC-MS

After precisely weighing 0.10 g of GQD powder, it was ultrasonicated for 30 min with 25 mL of a 50% methanol-water (*v*/*v*) solution. After a 10 min spin at 16,000 g, the collected samples were filtered via a 0.22 μm PTFE membrane. Dilute each reference substance at about 1 μg/mL using 50% methanol, respectively. And then for qualitative comparison under the same UPLC-MS conditions.

For quality control of GQD, an ACQUITY UPLCI-Class system was used with a Xevo XS quadrupole time of flight mass spectrometer (MS) (Waters, Milford, MA, USA). Chromatographic separation was performed using a Waters CORTECSC 18 Column (Waters, Milford, USA) (2.1 mm × 100 mm, 1.6 μm) at 45 °C. Mobile phase A comprised 0.1% formic acid in water, and mobile phase B was acetonitrile. The following described the gradient elution procedure: 5–10% B for 0–0.5 min, 10–13% B for 0.5–2.5 min, 13–15% B for 2.5–10 min, 15–15% B for 10–18 min, 15–20% B for 18–25 min, 20–50% B for 25–32 min. A 0.20 mL/min flow rate was observed. The optimal conditions for MS were as follows: ESI negative (−ve) and positive (+ve) modes, 500 °C dissolvent gas temperature, −2.5 kV and +3.0 kV capillary voltage, 150 °C source temperature, 800 L/h dissolvent gas flow, and 50 L/h cone gas flow. The range of the MS scan was 50–1500 m/z.

### 4.4. Collection and Evaluation of GQD Targets and Active Compounds

BATMAN-TCM (http://bionet.ncpsb.org.cn/batman-tcm/index.php) (accessed on 15 August 2023) was used to obtain the active components and target details of GQD [58]. The uniform gene names were obtained by using the UniProt database (https://www.uniprot.org/) (accessed on 15 August 2023) [59]. The screening criteria were targets with scores ≥ 20 were selected, and *p* ≤ 0.05 [60].

### 4.5. Collection of Disease Targets

Disease target data were retrieved using the GeneCards (https://www.genecards.org) and OMIM (https://www.omim.org) databases (accessed on 15 August 2023), with the keyword ‘Colitis-Associated Colorectal Cancer’ as the search query [61,62]. Among the targets obtained from both GeneCards and OMIM datasets, all of the targets related to CAC were included [63,64]. And the gene targets retrieved were a result of two merged disease databases, and all duplicate values were deleted.

### 4.6. Construction of PPI Network Diagram

The OmicShare platform (www.omicshare.com/tools) (accessed on 20 August 2023) was used to visualize Venny diagrams and display the intersecting genes that established connections between compounds and CAC target genes [65]. Simultaneously, a visual network map was developed using Cytoscape 3.9.1 software to identify core regulatory targets by inserting intersecting targets between GQD and CAC into the STRING database (https://cn.string-db.org/) (accessed on 20 August 2023), with biological species set to ‘Homo sapiens’. From this, a PPI was obtained and the TSV file format was downloaded [66]. Afterward, the original PPI information is input into Cytoscape 3.9.1 for visualization processing [67].

### 4.7. Constructing a Compound-Disease-Target-Pathway Network Diagram

The active ingredients of 4 herbs and their targets retrieved from the database were imported into Cytoscape 3.9.1 software for visualization. The Cytoscape 3.9.1 software can be updated with information regarding GQD-CAC targets, bioactive compounds, diseases, herbs, and signaling pathways. The network diagram illustrated the drug component targets and the network involved in the treatment of GQD for CAC [68,69].

### 4.8. Enrichment Analysis of GO and KEGG Pathways

The Database for Annotation, Visualization, and Integrated Discovery (DAVID) is the most widely used tool for analyzing the functional enrichment of genes [70]. The Benjamini-Hochberg method was utilized to rectify *p*-values, which were then adjusted for *p*-values. *p* ≤ 0.05 was designated as the significance term. To determine the function of key targets, we performed KEGG pathway enrichment analysis and GO enrichment analysis using DAVID [71].

### 4.9. Modeling and Intervention of AOM/DSS-Induced CAC in Mice

Thirty C57BL/6 mice (female, SPF grade, 6–8 weeks old) were procured from SLAC Laboratory Animal Technology Co., Ltd. (Shanghai, China). Its license number is SCXK (Shanghai) 2017-0005. The rearing conditions were constant temperature (20.8–24.3 °C) and humidity (47.2–67.2%), with a 12 h light-dark cycle. All mice were allowed free access to food and water. The bedding was changed every 2–3 days. Studies have reported that CAC induced by AOM/DSS in female C57BL/6 mice tends to produce more consistent and reproducible results [72]. The study protocol was approved by the Laboratory Animal Ethics Committee of Fujian University of Traditional Chinese Medicine (Ethics Approval No. 2W2022011 2022197). Following a week of acclimatization and feeding, the C57BL/6 mice were randomly divided into Control, AOM/DSS, and AOM/DSS + GQD groups. Each group consisted of ten mice. The Control group did not treat with AOM/DSS. Initially, the AOM was precisely weighed and dissolved in saline to achieve a concentration of 10 mg/mL. The solution was then thoroughly shaken to ensure uniformity [73]. With the exception of the Control group, mice in the AOM/DSS and AOM/DSS + GQD groups received intraperitoneal injections with 10 mg/kg AOM at the 1st day. From the 2nd day, the mice were given distilled water for 1 week. After 1 week, the DSS powder was accurately weighed and dissolved in drinking water to prepare a 2% DSS solution [74]. This solution was given for the daily consumption of the mice in both the AOM/DSS group and the AOM/DSS + GQD group for 1 week, after which distilled water was used for 2 weeks. Every 3 weeks was a DSS cycle, and 3 cycles were repeated [75]. Using body surface area-based equivalent dose calculation, 2 g/kg/day of GQD power was administered to the AOM/DSS + GQD group starting from the first day of drinking DSS water. Whereas the Control group and the AOM/DSS group received an equal volume of saline by gavage until the end of the experiment. Throughout the experiment, the mice’s body weight, the characteristics of their stools, and the presence of blood in their stools were all tracked daily.

### 4.10. DAI Evaluation

The changes in body weight, fecal occult blood, and fecal viscosity were tracked in each group of mice using the DAI scoring method. (1) Percentage of body weight loss: 0 ≤ body weight loss < 1%, 0 points; 1% ≤ body weight loss < 5%, 1 point; 5% ≤ body weight loss < 10%, 2 points; 10% ≤ body weight loss < 15%, 3 points; body weight loss ≥ 15%, 4 points. (2) Stool consistency: normal stool formation, 0 points; loose stool, 2 points; diarrhea, 4 points. (3) Fecal occult bleeding: no blood in the stool visible to the naked eye, 0 points; positive occult bleeding, 2 points; overt bleeding, 4 points; DAI = (weight loss score + fecal consistency score + fecal occult bleeding score)/3.

### 4.11. Sample Collection

At the end of the experiment, isoflurane was used to anesthetize the mice. After anesthetizing, the animals were euthanized via cervical dislocation. The length and weight of the descending colon were meticulously measured, and the quantity of tumors was noted. The collection of serum was used for ELISA experiments. The descending colon was dissected longitudinally. A portion of the tissue was used for HE staining and IHC analysis after fixation with paraformaldehyde (PFA). The remaining tissue was kept in a refrigerator at −80 °C for western blotting analysis.

### 4.12. Histopathological Analysis

The descending colon tissues underwent a 48 h fixation in PFA, processed by the gradient dehydration tissue embedding method, and were subsequently sectioned into multiple 4 μm thick sections. Subsequently, procedural dewaxing, rehydration, and HE staining were performed on the section. The histopathological examination was visualized by light microscopy (LEICA, Wetzlar, Germany).

### 4.13. IHC Analysis

Paraffin-embedded descending colonic tissue was subsequently sectioned into multiple 4 μm thick sections, IHC analysis was carried out using an IHC kit. The tissue sections were repaired with antigen retrieval and then washed three times with phosphate-buffered saline (PBS). Subsequently, the sections were incubated with an an endogenous peroxidase blocker at room temperature for 10 min. Afterward, they were rinsed three times with PBS and immersed in goat serum at room temperature for 1 h. Primary antibodies [Ki67 (1:200), F4/80 (1:200)] were added to the sections and incubated overnight at 4 °C. Following thorough washing, the sections were exposed to biotin-labeled IgG for 1 h at room temperature, followed by incubation with peroxidase for 1 h. DAB and hematoxylin was further used for staining. After being sealed in resin, 4 fields of each slice was captured under an light microscope (LEICA, Wetzlar, Germany). Each group comprised 4 mice, with one slice prepared for each mouse. The positive expression sites exhibited a characteristic brown-yellow granular appearance. The ImageJ analysis software (ImageJ Ver. 1.4.3.67, Maryland, USA) was then used to calculate the positive rate of protein [76].

### 4.14. Cell Culture

Mouse macrophage RAW264.7 cell line (CL-0190) were sourced from Procell Life Science & Technology Co., Ltd. (Wuhan, China). These cells were cultured at 37 °C in a humidified 5% CO_2_ incubator using Dulbecco’s Modified Eagle Medium (DMEM) supplemented with 10% Fetal Bovine Serum (FBS) and 1% Penicillin/Streptomycin (P/S). For experimental purposes, the cells were routinely passaged to the third generation.

### 4.15. Model of Inflammation In Vitro and GQD Treatment or the Combination of GQD and TAK242

The inflammatory model was developed using RAW264.7 cells that were induced with LPS in vitro. Cells were distributed into 6 groups: Control group, GQD group, LPS group, LPS + GQD 25 μg/mL, LPS + GQD 50 μg/mL, LPS + GQD 100 μg/mL group. The Control group was maintained in a blank medium, the GQD group was stimulated with GQD (50 μg/mL), and the LPS group were stimulated with LPS (1 μg/mL) to establish the inflammatory model. Following a 12 h GQD pre-treatment at the specified doses, the groups were stimulated with a medium containing 1 μg/mL LPS to initiate the inflammatory response.

To further evaluate the synergistic effect of GQD and TAK242 on the TLR4-related signaling pathways, the RAW264.7 cells were divided into Control group, LPS group, LPS + GQD (50 μg/mL) group, LPS + TAK242 (0.1 μM) group, LPS + GQD (50 μg/mL) + TAK242 (0.1 μM) group. The process of administration was as described above.

### 4.16. Cell Viability Assay

To evaluate the impact of GQD on cell activity, the cytotoxicity assay was performed via a CCK8 kit. A density of approximately 1 × 10^5^ cells/mL RAW264.7 cells were allowed to grow in a 96-well plate and exposed to varying concentrations of GQD for 24 h. Another 96-well plate was pretreated for 12 h with various doses of GQD (0, 25, 50, 100 μg/mL) before being stimulated for 12 h with 1 μg/mL LPS. Following treatment, the 10 μL of CCK8 reagent was added into each well for 2 to 4 h. The absorbance at 450 nm was measured via a Microplate Reader (Thermo Fisher Scientific, USA).

### 4.17. Quantitative Real-Time PCR Determination of Cytokines

Total RNA from RAW264.7 cells was extracted using the Trizol reagent kit. RNA concentration was determined using a NanoDrop2000 ultra-micro analyzer (Thermo Fisher Scientific, USA). Isolated RNA was employed as a template for cDNA synthesis via the HiScript 1st Strand cDNA Synthesis kit. The SYBR^®^ Select Master Mix kit and the primers for IL-1β, IL-6, and TNF-α were used to amplify cDNA. GAPDH served as an internal control. The transcriptional levels of IL-1β, IL-6 and TNF-α, were quantified using the 2^−ΔΔCT^ method [77]. The primers are shown in Table 1.

### 4.18. Immunofluorescence Assay

After fixing the cells for 15 min with 4% PFA, they were exposed to 1% Triton for 5 min at room temperature. After washing with PBS, the cells were kept in 5% BSA solution for 2 h to block them. Subsequently, the cells were exposed to the primary antibodies [NF-κB (1:200), IRF3 (1:200)] and incubated overnight at 4 °C. The cells were then incubated with the respective secondary antibody (1:200) at room temperature for 1 h, and then DAPI was added to stain the cells. Finally, confocal laser scanning (LEICA, Wetzlar, Germany) was used to observe and photograph.

### 4.19. Detection of Inflammatory Factors via ELISA

The serum of mice and the supernatant of cells were both collected for the assessment of IL-1β, IL-6, and TNF-α levels. Standard wells and sample wells were set up. To each standard well, 50 μL of standards with varying concentrations was added. For the sample wells, 10 μL of the test sample was first added, followed by 40 μL of sample diluent. The blank well was left untreated. Except for the blank well, 100 μL of the detection antibody labeled with horseradish peroxidase was added to each of the other wells. After sealing the plate, the wells were incubated in an incubator at 37 °C for 60 min. The liquid was then discarded, and the wells were patted dry. The washing liquid was added to each well. After standing for 1 min, the washing liquid was shaken out, and the wells were patted dry again. This operation was repeated 5 times. 50 μL each of substrate A and substrate B was added to each well, and they were incubated in an incubator at 37 °C in a dark environment for 15 min. 50 μL of stop solution was added to each well, and the absorbance of each well was measured at 450 nm in a Microplate Reader (Thermo Fisher Scientific, USA) [78].

### 4.20. Western Blotting Analysis

Proteins from the colon tissue and cell samples were extracted using RIPA containing protease inhibitors and phosphatase inhibitors. Protein quantification was determined by the BCA method. Diluted the sample to be tested, added the working solution, conducted the reaction under the same conditions, measured the absorbance, and substituted it into the curve to calculate the protein concentration of samples. A total of 40 µg protein was separated on 10% SDS-PAGE gel and transferred the proteins onto PVDF membrane by electroblotting. Further, the membranes were blocked for 2 h using 5% skim milk. The primary antibodies [TLR4 (1:1000), MyD88 (1:5000), TRIF (1:1000), p-IRF3 (1:1000), IRF3 (1:1000), p-IκBα (1:1000), IκBα (1:1000), p-P65 (1:1000), P65 (1:1000), β-actin (1:10,000), GAPDH (1:10,000), Vinculin (1:5000)] were added to the membranes overnight at 4 °C, respectively. The next day, the PVDF membrane was washed 4 times with TBST, each time for 5 min. After adding the corresponding mouse secondary antibody or rabbit secondary antibody (both 1:10,000), the membrane was blocked at room temperature for 1 h, followed by washing the membrane 3 times with TBST, each time for 5 min. The images of the membranes were examined via an enhanced chemiluminescence (ECL) detection. Finally, the ImageJ analysis software (ImageJ Ver. 1.4.3.67, USA) was then used to statistically analyze the gray values of the bands [79]. GAPDH, β-actin and Vinculin served as the internal control. The relative protein expression was quantified by normalizing the Control group as 1.

### 4.21. Statistical Analysis

Data analysis was carried out by means of SPSS software (SPSS Ver. 22.0, USA) and GraphPad Prism Software (GraphPad Prism Ver. 8.0.2, USA). The measurement data, which were expressed as mean ± standard deviation. The normality of the data distribution was assessed via either the Shapiro-Wilk test or the Kolmogorov-Smirnov test, followed by one-way ANOVA and Tukey’s HSD post hoc test for pairwise comparisons. If variances were unequal, a non-parametric Kruskal-Wallis test was used, accompanied by Dunn’s post hoc test for pairwise comparisons. Statistical significance was denoted as *p* ≤ 0.05.

## 5. Conclusions

Our study revealed that GQD attenuated the symptoms, reduced tumor incidence, and improved the survival rate in mice subjected to AOM/DSS. Furthermore, GQD suppressed inflammatory response via suppressing TLR4-related NF-κB and IRF3 signaling pathways. These results provide a more reliable scientific infrastructure for the preclinical use of GQD.

## Figures and Tables

**Table 1:** Primer sequences for RT-PCR.

Genes	Forward Sequence (5′-3′)	Reverse Sequence (5′-3′)
GAPDH	ACGGCAAGTTCAACGGCACAG	GAAGACGCCAGTAGACTCCACGAC
IL-1β	GAAATGCCACCTTTTGACAGTG	TGGATGCTCTCATCAGGACAG
IL-6	CTGCAAGAGACTTCCATCCAG	AGTGGTATAGACAGGTCTGTTGG
TNF-α	GCCGATGGGTTGTACCTTGT	TCTTGACGGCAGAGAGGAGG

## Data Availability

Data is contained within the article.
